# Network Proximity Analysis Deciphers the Pharmacological Mechanism of Osthole against D-Galactose Induced Cognitive Disorder in Rats

**DOI:** 10.3390/molecules29010021

**Published:** 2023-12-19

**Authors:** Xue Wang, Xiaomei Fu, Xiurong Luo, Yiyi Lai, Chuipu Cai, Yanfang Liao, Zhao Dai, Shuhuan Fang, Jiansong Fang

**Affiliations:** 1 Science and Technology Innovation Center, Guangzhou University of Chinese Medicine, Guangzhou 510405, China; wangxue1666@163.com (X.W.); xmf112311@163.com (X.F.); xiurongluo168@163.com (X.L.); 13667066510@163.com (Y.L.); liaoyanfang211@163.com (Y.L.); 20212110070@stu.gzucm.edu.cn (Z.D.); 2 Division of Data Intelligence, Department of Computer Science, Key Laboratory of Intelligent Manufacturing Technology of Ministry of Education, College of Engineering, Shantou University, Shantou 515063, China; caichuipu@gzucm.edu.cn

**Keywords:** Alzheimer’s disease, cognitive disorder, Osthole, neuronal apoptosis, neuroinflammation, network proximity

## Abstract

Osthole, a natural coumarin found in various medicinal plants, has been previously reported to have neuroprotective effects. However, the specific mechanism by which Osthole alleviates dysmnesia associated with Alzheimer’s disease (AD) remains unclear. This study aimed to investigate the neuroprotective properties of Osthole against cognitive impairment in rats induced by D-galactose and elucidate its pharmacological mechanism. The rat model was established by subcutaneously injecting D-galactose at a dose of 150 mg/kg/day for 56 days. The effect of Osthole on cognitive impairment was evaluated by behavior and biochemical analysis. Subsequently, a combination of in silico prediction and experimental validation was performed to verify the network-based predictions, using western blot, Nissl staining, and immunofluorescence. The results demonstrate that Osthole could improve memory dysfunction induced by D-galactose in Sprague Dawley male rats. A network proximity-based approach and integrated pathways analysis highlight two key AD-related pathological processes that may be regulated by Osthole, including neuronal apoptosis, i.e., neuroinflammation. Among them, the pro-apoptotic markers (Bax), anti-apoptotic protein (Bcl-2), the microgliosis (Iba-1), Astro-cytosis (GFAP), and inflammatory cytokines (TNF-R1) were evaluated in both hippocampus and cortex. The results indicated that Osthole significantly ameliorated neuronal apoptosis and neuroinflammation in D-galactose-induced cognitive impairment rats. In conclusion, this study sheds light on the pharmacological mechanism of Osthole in mitigating D-galactose-induced memory impairment and identifies Osthole as a potential drug candidate for AD treatment, targeting multiple signaling pathways through network proximity and integrated pathways analysis.

## 1. Introduction

Alzheimer’s disease (AD) is one of the main syndromes of senile dementia, with memory loss and cognitive dysfunction as the main clinical manifestations. It is estimated that the number of people aged 65 and older with AD is projected to reach 7.2 million by 2025 [1]. Characterized by extracellular amyloid-beta (Aβ) deposits and intracellular neurofibrillary tangles (NFTs) composed of highly phosphorylated tau proteins, AD is a type of disease with complex pathophysiological processes. The pathogenesis of AD is relevant to the complex interplay between the disruption of synaptic homeostasis and the malfunction in the highly correlated endosomal/lysosomal clearance pathway, and so on [2]. Unfortunately, the search for efficient drugs to impede or decelerate the progression of this disease has been unfruitful owing to the intricate and heterogeneous nature of the disease [3,4]. Considerable research and continuous endeavors are committed to the development of effective treatments that can tackle both the symptoms and the underlying progression of AD. These initiatives involve the exploration of diverse mechanisms, including but not limited to the reduction of amyloid plaque accumulation, targeting tau protein, addressing neuroinflammation, and investigating alternative therapeutic pathways.

Osthole, named also as osthol, is a naturally occurring coumarin derivative found in a variety of Chinese medicinal herbs, such as *Cnidium monnieri* and *Angelica pubescents* [5]. Previous studies have revealed that Osthole exerts extensive pharmacological activities, such as anti-inflammatory, neuroprotective and antioxidant activities, and immunity enhancement [6]. For instance, Osthole has been shown to promote neural stem cell proliferation and neuronal differentiation while inhibiting apoptosis through the Wnt/*β*-catenin signaling pathway [7]. Additionally, Osthole improves synaptic plasticity in AD model rats by regulating glutamate levels [8]. Moreover, Osthole exhibits the ability to regulate oxidative stress, reduce glutamate-induced apoptosis, and protect neurons in both in vitro and in vivo models of AD [9]. However, despite these findings, the precise neuroprotective and regulatory mechanisms of Osthole in alleviating AD-associated dysmnesia are not yet fully understood due to the complex nature of the pathological mechanisms underlying AD.

As a rapidly developing interdisciplinary field, network medicine offers a holistic approach to integrating vast amounts of multi-omics data in order to redefine human diseases and therapeutic strategies. It presents a comprehensive framework that allows for the synthesis of diverse data sources, enabling a fresh perspective on understanding and addressing complex medical conditions [10,11]. Due to the inherent complexity of AD, there are common underlying mechanisms and intermediate endophenotypes between AD and other diseases. As an example, six typical endophenotype networks have been proposed in AD: (i) amyloidosis, (ii) tauopathy, (iii) neuroinflammation, (iv) mitochondrial dysfunction, (v) vascular dysfunction, and (vi) lysosomal dysfunction [12]. Recently, we have applied an endophenotype-based approach to repurpose sildenafil as a candidate drug for AD [13], and explored the neuroprotective mechanisms of Medicarpin in scopolamine-induced cognitive impairment mice [14]. Thus, AD-related endophenotype modules can be effectively employed to characterize pathogenesis and drug therapeutic mechanisms for AD.

**Figure 1:**
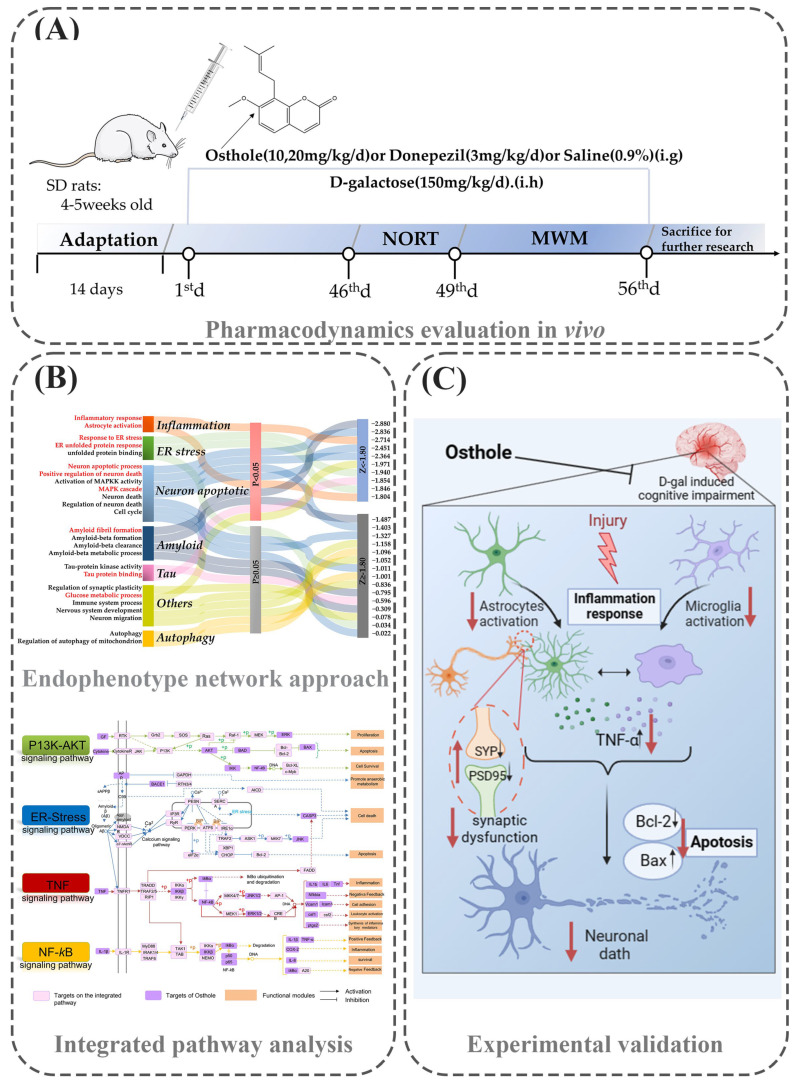
Endophenotype network-based mechanism exploration framework for Osthole versus AD. (**A**) The anti-AD effects of Osthole in enhancing spatial learning and memory in D-galactose- treated rats by biochemical analysis and behavioral test. (**B**) In silico identification of anti-AD mechanisms for Osthole by network proximity prediction and drug-target network analysis. (Red font represents endophenotype modules with significant proximity degrees) (**C**) Experimental validation of the findings of network-based prediction (Red arrows indicate regulatory effects of Osthole (upwards for positive regulation and downwards for negative regulation). Black arrows represent impairing effects of D galactose-induced cognitive impairment model (upwards for up-regulation and downwards for down-regulation)). Created with BioRender.com. Abbreviations: Sprague Dawley rats (SD rats); New object recognition test (NORT); Morris water maze test (MWM); D-galactose (D-gal).

In this work (Figure 1), firstly, Osthole demonstrated its ability to enhance spatial learning and memory in rats induced with D-galactose (D-gal), indicating its potential as an anti-AD agent. To investigate the mechanisms of Osthole against AD, we employed an endophenotype network strategy that incorporated AD-related endophenotype modules, a human protein–protein interactome, and a network of Osthole targets. Specifically, we utilized network proximity prediction to assess the network distance between the AD-related endophenotype module and the Osthole target network. Subsequently, we constructed integrated pathways to identify the biological pathways regulated by Osthole targets that are associated with AD. Finally, we validated this proposed pharmacological mechanism through experimental techniques.

## 2. Results

### 2.1. Osthole Alleviates D-Galactose-Induced Cognition and Synaptic Dysfunction

**Figure 2:**
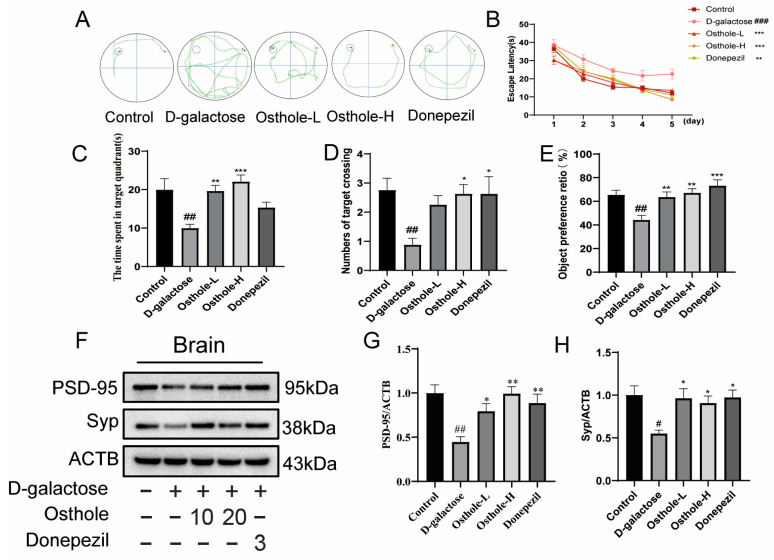
Osthole ameliorates D-galactose-induced memory and spatial cognition and synaptic dysfunction in rats. (**A**) Representative trajectories; (**B**) escape latency in seconds to reach the hidden platform during training (5 days) along with; (**C**) the time spent in the target quadrant; (**D**) numbers for target crossing; (**E**) object preference ratio; Western blot analysis of synapse function-related proteins PSD-95 (**F**,**G**) and Syp (**F**,**H**) in brains. For behavioral studies, *n* = 8; (**B**) multivariate ANOVA with Bonferroni; (**C**–**E**) analyzed by ANOVA with Dunnett’s multiple comparisons test); for Western blot analysis; (**G**,**H**) analyzed by ANOVA with Dunnett’s multiple comparisons test, *n* = 4). ^#^ *p* < 0.05, ^##^ *p* < 0.01, ^###^ *p* < 0.001 vs. Controls. * *p* < 0.05, ** *p* < 0.01, *** *p* < 0.01 vs. D-galactose-treated rats.

Cognitive dysfunction and memory deficits are the primary early symptoms of AD. Previous studies have shown that long-term high-dose injection of D-gal can lead to neuroinflammation, neuron damage and cognitive dysfunction in rats [15,16]. The Morris water maze test (MWM) and new object recognition test (NORT) were applied for assessment of whether Osthole could improve spatial learning and memory impairment. Eight rats in each experimental group were randomly selected for behavioral testing. The representative swimming trajectory and the time of escape (seconds) to the hidden platform during the training period (5 days) is shown in Figure 2A,B. Time for the D-gal group from the second day of swimming training was significantly longer than that for the control group, while escape latency was significantly reduced in the Osthole treatment group and Donepezil group. Similarly, after training, the hidden platform was removed and the last day of testing was performed, showing that the Osthole group and Donepezil group remained in the target quadrant longer than the D-gal group rats in Figure 2C. In addition, the number of platform crossings was significantly increased in the Osthole and Donepezil groups compared with the D-gal group in Figure 2D, showing that Osthole can improve spatial learning disabilities induced by D-gal. The results of the NORT are shown in Figure 2E. Compared with the control group, the level of discrimination index of the D-gal treatment group showed a significant decrease, whereas Osthole-treated group or Donepezil-treated group significantly improved performance in the recognition index.

Western blot analysis of synaptophysin (Syp) and postsynaptic density protein (PSD95), the presynaptic protein associated with memory, was significantly reduced in the brains of D-gal-treated rats compared to control rats. Interestingly, Osthole and Donepezil significantly up-regulated the protein of PSD95 and Syp in the brains of D-gal -induced rats (Figure 2F–H), all of this indicating that Osthole exerted beneficial effects on D-gal-induced learning and memory disorders.

### 2.2. Construction of the Drug-Target Network for Osthole

**Figure 3:**
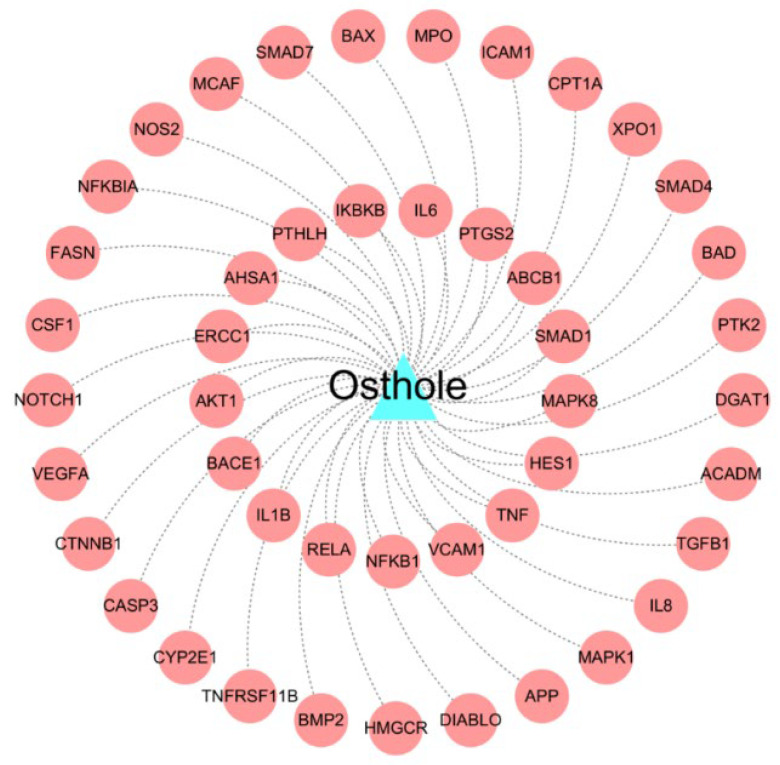
The drug-target interactions (DTIs) of Osthole.

The constructed drug-target interactions network for Osthole contains 46 known experimentally validated targets (Figure 3 and Supplementary Table S1). Multiple pieces of evidence suggest that Osthole has the potential to exert anti-AD effects through the regulation of these targets. For instance, the *β*-site APP catabolizing enzyme (BACE) has garnered attention as a possible therapeutic target and biomarker for AD. Its contribution lies in the production of Aβ peptides [17]. Recent research has shown that focused blocking of BACE-1 in microglia yields better results in enhancing cognitive abilities [18]. Caspase-3, a regulator involved in controlling cell death (apoptosis), was discovered to be responsible for causing synaptic dysfunction in mice models with AD in a prior investigation [19]. Overall, the DTIs network presents valuable insights that aid in understanding the precise targeting and combating of AD by Osthole, thereby exposing its underlying therapeutic mechanism.

### 2.3. Construction of Endophenotype Network for AD

**Figure 4:**
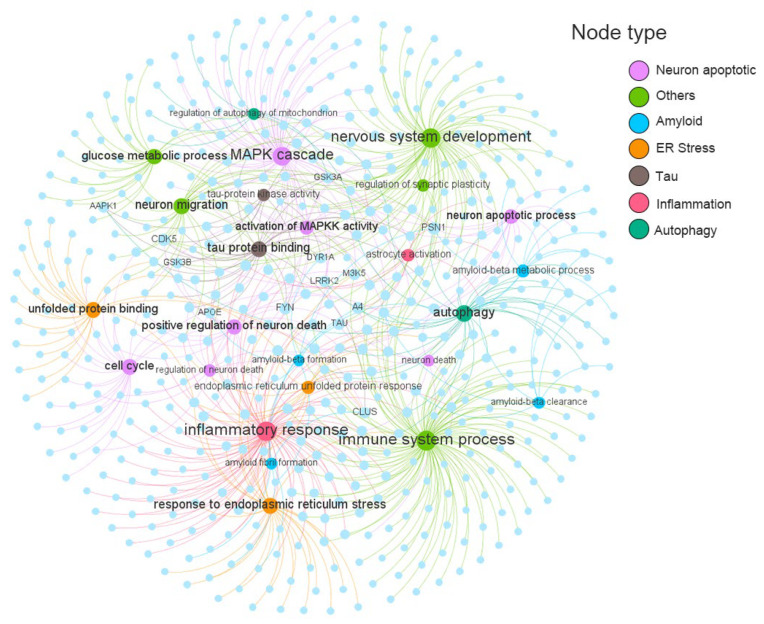
AD target-endophenotype modules network. Labels showing the 25 AD pathology modules and the top 13 targets with the highest degree values. The label font size and node size correspond to the degree. The 25 pathology modules are divided into seven categories and displayed in different colours.

Based on previous studies, these endophenotype modules can be grouped into seven major classifications, including amyloidosis, neuron inflammation, neuron apoptosis, endoplasmic reticulum stress, tauopathy, and autophagy [14]. The detailed gene catalog for these endophenotype modules can be found in Supplemental Material Table S2. As shown in Figure 4, the AD-specific target-endophenotype modules network contains 703 target- pathological process pairs, connecting seven different types of modules consisting of 25 AD pathological processes and 486 genes. Four of the 25 pathology modules have a target number (*n*) exceeding 50, including immune system process (*n* = 115), inflammatory response (*n* = 96), nervous system development (*n* = 83), and MAPK cascade (*n* = 67). Eight of the 486 Ad targets simultaneously target more than five pathological modules (D): PSN1 (D = 8), CDK5 (D = 7), A4 (D = 7), FYN (D = 6), CLUS (D = 6), LRRK2 (D = 5), TAU (D = 5), M3K5 (D = 5). These genes have been shown in previous studies to be involved in a variety of pathological mechanisms associated with AD. For example, dysregulation of cyclin-dependent kinase 5 (CDK5) triggers the formation of A*β* plaques and NFTs, synaptic damage, mitochondrial dysfunction, and neuronal apoptosis, which are among a series of AD-related pathological events [20].

### 2.4. Network Proximity Identified Potential AD Pathological Mechanisms Regulated by Osthole

**Figure 5:**
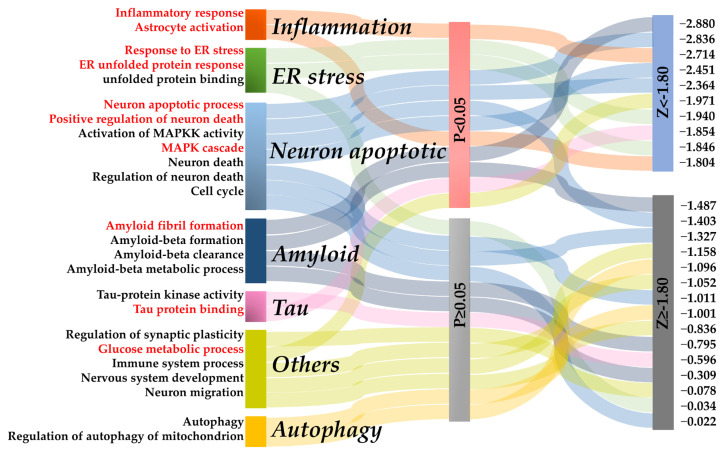
A global view of 25 AD endophenotype modules identified and the correlation among the Osthole-target network by network proximity. AD endophenotype modules with a *p* value < 0.05 and Z < −1.8 were regarded as significant (highlighted in red font).

Osthole significantly attenuated spatial learning and memory deficits in rats with D-gal-induced memory impairment. Next, the therapeutic mechanism of Osthole on AD was discovered using the network proximity approach by measuring the network distance between AD phenotype modules and the target network of Osthole. It was hypothesized that it was within or near a specific disease endophenotype module in the human interactome network that the target network of Osthole should be found, and the therapeutic effect of Osthole on AD is contributed to by targeting multiple AD-related endophenotype mechanisms. As shown in Figure 5, after setting *p* < 0.05 and Z < −1.8 as the network proximity scoring thresholds, 10 AD-related pathways were identified to be significantly correlated with Osthole.

Neuroinflammation is a vital pathological factor in AD [21], the network prediction revealing that Osthole might regulate the inflammatory response (Z = −2.714). Meanwhile, neuronal apoptosis occurs extensively during development and pathology, including AD [22], and we predicted three apoptosis-related pathways, including positive regulation of neuron death (Z = −2.364), MAPK cascade (Z = −2.451) and neuron apoptotic process (Z = −2.880). Besides, ER stress, including responses to ER stress (Z= −1.940) and ER unfolded protein response (Z = −1.846), also emerge as significant. Increasing evidence has demonstrated that ER stress was involved in several brain pathological processes observed in AD, such as *β*-amyloid production, tau phosphorylation, inflammation and cell death, and thus plays an essential role in the pathogenesis of AD [23]. Our prediction suggested that neuroinflammation, ER stress and apoptosis could be modulated by Osthole to exert anti-AD outcomes, warranting further analysis and validation.

### 2.5. Integrated Pathway Map Analysis

**Figure 6:**
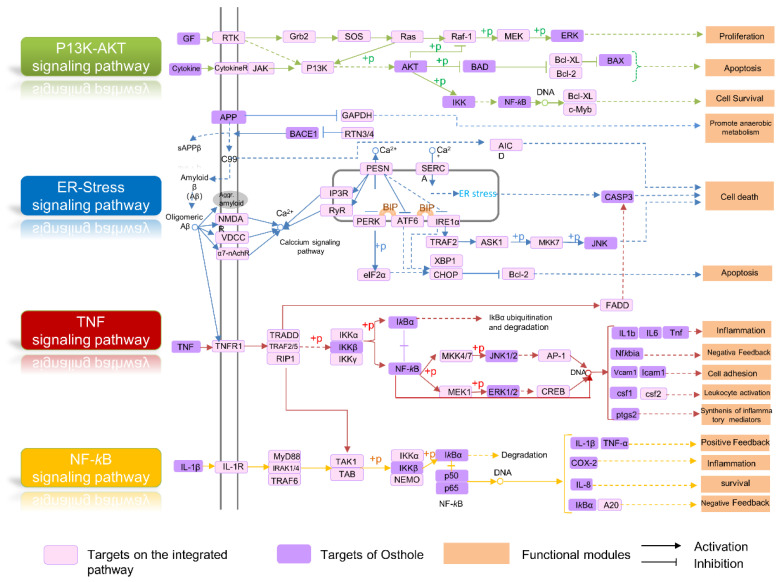
AD-integrated pathway and functional modules: The pathway integration was based on the experimentally validated targets of Osthole and relative pathways in the KEGG database.

According to the network-based analysis, an integrated pathway map was drawn via mapping drug targets of Osthole into the pathways that were directly related to the pathological process of AD. As depicted in (Figure 6), this map consisted of four pathways, including the PI3K/AKT signaling pathway, ER stress-related signaling pathways, TNF signaling pathway and NF-*κ*B signaling pathway. Here, three representative functional modules involved in the map are selected to illustrate the potential mechanism of Osthole in the treatment of AD.

#### 2.5.1. Apoptosis Module

Apoptosis is a programmed cell death that occurs when the organism is damaged or affected by various factors [24,25]. In the brains of AD patients, most neuronal death is mediated by apoptosis and apoptotic neuronal death, which were likely to be important characteristics of AD [26]. PI3K-AKT is a major cell survival pathway that regulates apoptotic mechanisms and proteins associated with anti-apoptotic factors via phosphorylation [27,28]. As shown in Figure 6, Osthole could act on multiple targets in the PI3K-AKT signaling pathway, as well as proteins highly associated with apoptosis (e.g., BCl-2, CASP3), suggesting its potential anti-AD mechanism mediated by apoptosis.

#### 2.5.2. Neuroinflammation Regulation Module

Neuroinflammation tends to be a chronic process that fails to resolve by itself and is recognized to be one of the driving forces for neurodegeneration [29]. The interaction of Aβ with microglia and astrocyte promotes the production of inflammatory cytokines, such as, TNF-α, IL-1 and IL-6, which further increases the level of activated microglia and Aβ, thereby promoting the inflammatory progression of AD [30,31]. In addition, activation of NF-κB promotes the expression of inflammatory cytokines, such as Cyclooxygenase-2 (COX-2), TNF-α and IL-1β [32,33]. Figure 6 shows that Osthole may exert potential anti-inflammatory effects in AD treatment by affecting key proteins in the TNF signaling pathway and NF-κB signaling pathway.

#### 2.5.3. Endoplasmic Reticulum Stress Regulation Module

Several studies have reported that endoplasmic reticulum (ER) stress plays an integral role in the pathogenesis of AD [23]. Accumulating evidence suggests that the Aβ oligomer disrupts Ca^2+^ homeostasis and increases ROS production, leading to oxidative stress and caspase-3-related cell death [34]. Neurological dysfunction and cell death are commonly associated with ER stress, which is initiated by the accumulation of misfolded proteins and disruption of intracellular calcium equilibrium [35]. Figure 6 shows that Osthole targets several proteins implicated in the Aβ- associated ER stress signal pathway, including BACE1, APP, JNK, and CAPS3, indicating that its underlying anti-AD mechanism might be related to the regulation of ER stress-induced apoptosis.

### 2.6. Osthole Inhibited D-Galactose-Induced Neuron Loss and Neuron Apoptosis

**Figure 7:**
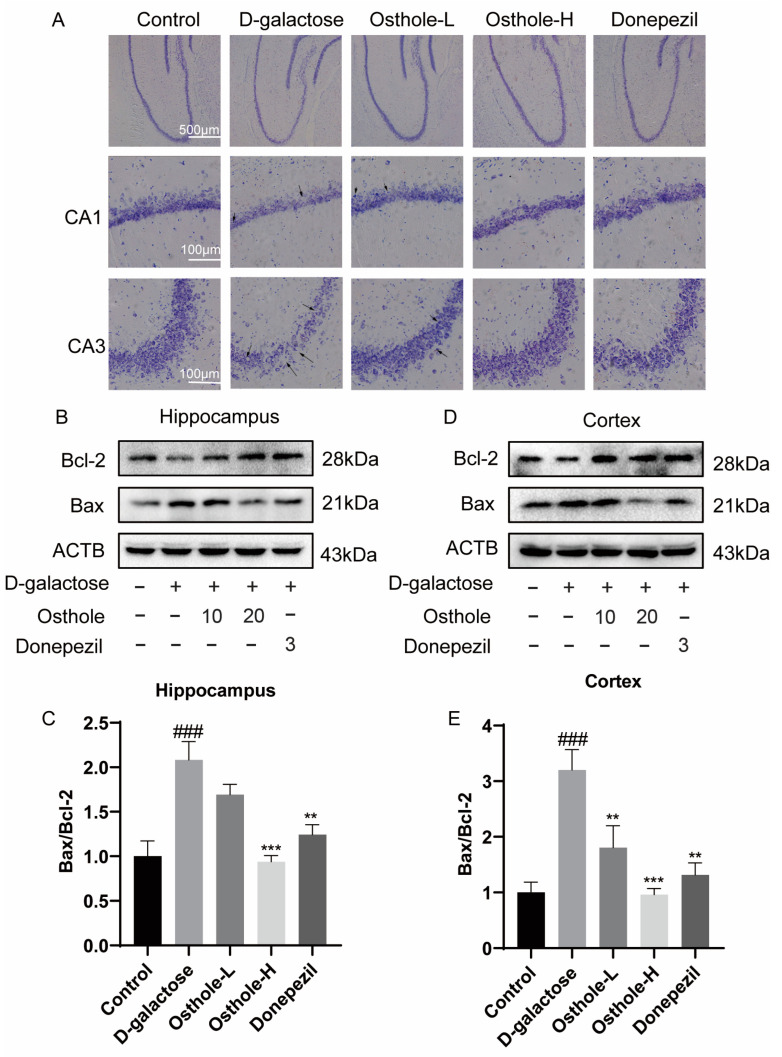
Osthole decreases neuron loss and neuron apoptosis induced by D-galactose. (**A**) Nissl staining images of hippocampal CA1 and CA3 regions (number of experiments = 3. Scale bar: 100 µm; the arrows indicate disorganization of hippocampal Nissl bodies, characterized by blurred edges, reduced numbers of Nissl bodies, and partial vacuolization). Representative western blot analysis of the effect of Osthole and Donepezil on Bax, Bcl-2 level in hippocampus (**B**,**C**) and cortex, (**D**,**E**) and on rats with or without D-galactose. (Data represent mean values ± SEM and were analyzed by Dunnet’s test after one-way analysis of variance, *n* = 4). ^###^ *p* < 0.01 vs. Controls. ** *p* < 0.01, *** *p* < 0.001 vs. D-galactose-treated rats.

Our network analysis indicated that Osthole had a positive impact on mitigating the learning and memory impairments caused by D-gal. This could potentially be achieved through the regulation of neuronal apoptosis. In this study, we sought to determine if the observed enhancement in rat’s learning and memory abilities was indeed linked to a reduction in neuron loss and to mediating neuronal apoptosis-related pathways in both the hippocampus and cerebral cortex. As evident from the representative images of Nissl staining in Figure 7A, the hippocampal Nissl bodies in the CA1 and CA3 regions of the D-gal group exhibited disorganization, characterized by blurred edges, a decreased number of Nissl vesicles, and partial vacuolation. Conversely, the Osthole or Donepezil treatment group displayed a noticeable increase in the count of hippocampal Nissl bodies, along with alignment, well-defined edges, and reduced vacuolization (Figure 7A).

To investigate the expression of pro-apoptotic and anti-apoptotic markers, Western blotting was performed. The research findings consistently demonstrate a significant upregulation in the expression of Bax protein, a pro-apoptotic factor, within both the hippocampus and cortex of D-gal-treated rats. Concurrently, there is a pronounced downregulation in the expression of Bcl-2 protein, an anti-apoptotic molecule, in these regions. As a result, these alterations culminate in D-gal-induced cell death and the initiation of apoptotic processes. Conversely, treatment with either Osthole or Donepezil effectively decreased the elevated expression of Bax while upregulating the expression of Bcl-2 in both the hippocampus (Figure 7B,C) and cortex (Figure 7D,E) of rats. These results provide evidence that Osthole has the potential to mitigate cell death and neuronal apoptosis induced by D-gal in rats.

### 2.7. Osthole Inhibited D-Galactose-Induced Neuroinflammation

**Figure 8:**
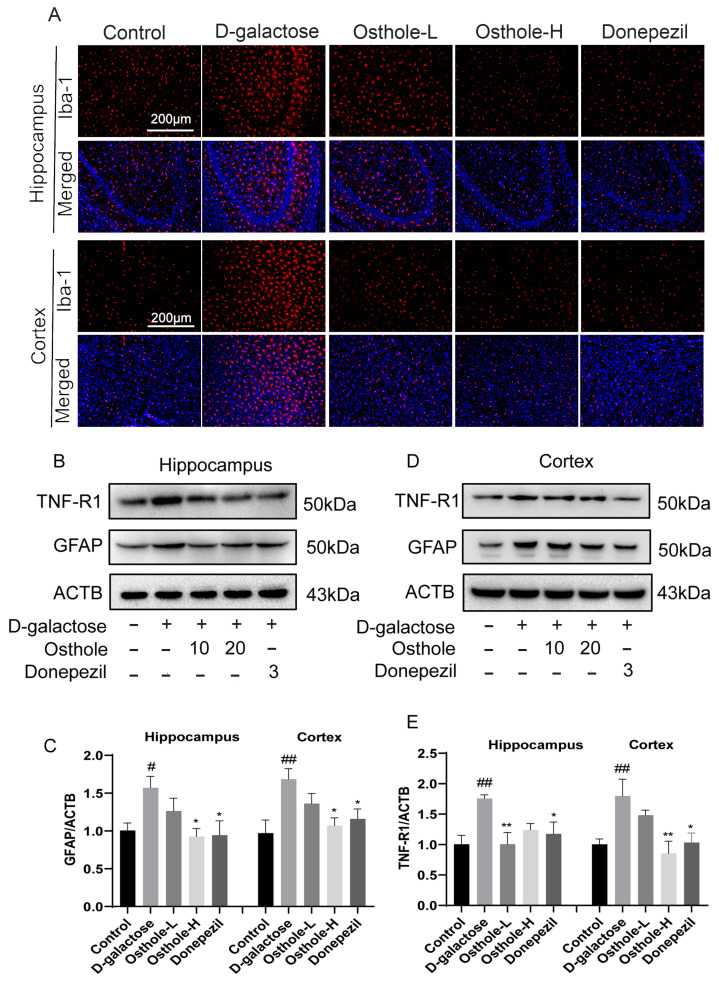
Osthole inhibited D-galactose-induced neuroinflammation. (**A**) The immunofluorescence images represent the immunoreactivity of Iba-1 (activated microglia) in the cortex and hippocampus (CA-3), *n* = 3. Scale bar: 200 μm. Western blot analysis of GFAP, TNF-R1 in the hippocampus (**B**,**C**) and cortex (**D**,**E**) from D-galactose-induced rats. (Data represent mean values ± SEM and were analyzed by Dunnet’s test after one-way analysis of variance, *n* = 4). ^#^ *p* < 0.05, ^##^ *p* < 0.01 vs. Controls. * *p* < 0.05, ** *p* < 0.01 vs. D-galactose-treated rats.

According to our network analysis, the anti-AD mechanism of Osthole may be also related to the regulation of neuroinflammation. To analyze the implication of Osthole on the expression of neuroinflammation, we performed immunoblotting and immunofluorescence to observe the levels of Iba-1 (activated microglia), GFAP (astrocyte activation), and TNF-R1 (tumor necrosis factor receptor 1). As indicated in Figure 8A and Figure S1, there was a notable rise in the number of Iba-1-positive cells in the hippocampus and cortex of rats within the D-gal group, in contrast to the control group. Interestingly, both the Osthole-H group and Donepezil group showed a substantial decrease in the number of Iba-1-positive cells in both the hippocampus and cortex, when compared to D-gal group alone. Figure 8B–E demonstrate a noteworthy increase in the expressions of GFAP and TNFα1 in the hippocampus and cortex of the D-gal group, in contrast to the control group. Interestingly, these expressions were down-regulated in both the Osthole and Donepezil groups, indicating that Osthole has the potential to regulate glial (especially microglia and astrocytes) cell activation and neuroinflammation induced by D-gal.

## 3. Discussion and Conclusions

Alzheimer’s disease is a progressive disorder characterized by cognitive dysfunction and memory loss. This condition advances over time, leading to the loss of neurons, changes in glial cells, and the onset of dementia [36]. Recent research has highlighted that the pathological changes associated with AD can occur up to three decades before symptoms appear. Additionally, the importance of controlling environmental risk factors, embracing healthy lifestyles, conducting early screening for AD using reliable biomarkers and utilizing natural multi-targeted drug therapies has been underscored as critical in effectively managing the disease [37,38]. D-galactose is an aldohexose, a reducing sugar that occurs naturally in the body and in many foods. Chronic systemic administration of D-galactose in animals may induce brain aging, which is similar to human brain ageing in many ways, including cognitive deficits, mitochondrial dysfunction, neuronal degeneration and apoptosis [39,40]. The D-galactose-induced cognitive dysfunction model in rats is widely accepted as a reliable methodology for studying memory impairment, a prominent feature of AD.

Our study found that Osthole, a natural coumarin, could alleviate memory and synaptic dysfunction induced by D-galactose in SD rats. An endophenotype network strategy was utilized that assembled AD-related endophenotype modules and target information for Osthole in the human protein interactome to comprehensively identify the underlying neuroprotective mechanism of Osthole against AD. We then applied network analysis, including network proximity analysis and integrated pathway analysis, to decipher the potential mechanisms of action of Osthole. Based on our network analysis, it has been predicted that Osthole has the ability to regulate multiple signaling pathways involved in countering AD. These pathways primarily include the PI3K/AKT signaling pathway, ER stress-related signaling pathways, TNF signaling pathway, and NF-*κ*B signaling pathway. The key aspect of these pathways lies in their regulation of neuronal apoptosis and neuroinflammation. According to the guidance provided by network-based prediction, we proceeded to conduct in vivo experiments to explore the underlying pharmacological mechanism of Osthole in neuroprotection. Our experimental results demonstrated a clear inhibition of D-galactose-induced neuronal loss by Osthole, as evidenced by Nissl staining. The Bcl-2 family is known for its pivotal role in apoptosis regulation, with the Bax gene being a pro-apoptotic member of this family. The balance between Bax and Bcl-2 determines the fate of apoptosis [41,42]. Notably, our study observed a significant increase in the Bax to Bcl-2 ratio in the D-galactose group. However, treatment with Osthole effectively reduced this ratio, indicating its potential neuroprotective effect through modulation of Bcl-2 and Bax interactions.

Microglia are macrophages of the central nervous system and play an important role in neuroinflammation [31]. Similar to microglia, there is substantial evidence that reactive astrocytes induce neuroinflammatory as well as neurotoxic effects, and that there is mutual regulation between astrocytes and microglia. In pathological conditions, over-activated microglia produce many inflammatory cytokines or act directly on nervous tissues to aggravate inflammatory reactions in conjunction with reactive astrocyte [29,43]. TNF-α is a component of neuropathological changes, one of the up-regulated immune genes in AD. Chronic exposure to inflammatory stimulation of macrophages can induce neurotoxic progression and induce neurotoxic astrocyte [30]. In addition, reactive microglia stimulate tau pathology in a cell-autonomous manner. TNF-R1 belongs to the superfamily of tumor necrosis factors and is one of the most important TNF-α receptors, which is expressed in most tissues and crosslinks with TNF to produce a pro-inflammatory response. The results of immunofluorescence and immunoblotting of Iba1, GFAP and TNF-R1 suggested that Osthole could down-regulate a D-galactose-induced inflammatory response.

Our work presents a number of innovations and limitations. Firstly, we employed a network medicine approach to systematically investigate the therapeutic mechanism of Osthole against AD. This finding further validates the reliability of the endophenotype network and network proximity approaches in studying the mechanism of action of herbal monomers. However, it is important to acknowledge that there may still be limitations in the drug target network of Osthole due to incomplete experimental validation of targets. Secondly, although our endophenotype network has covered targets involved in common AD pathological hypotheses, there is still incompleteness in the endophenotype module. This is primarily due to the fact that the complex pathogenesis of AD has not been fully elucidated. Furthermore, our predictions based on the network distance method and target pathway analyses have highlighted several important pathological processes regulated by Osthole. However, only the neuronal apoptotic and neuroinflammatory functions have been experimentally validated, and primary targets as well as reverse validation are lacking. The remaining predicted mechanisms, such as ER stress, A*β* or tau-related pathways, glutamate metabolism, and autophagy, warrant further investigation in subsequent studies.

Collectively, in the combination of network proximity prediction and in vivo validation, this study demonstrated that Osthole could improve D-galactose-induced cognitive and memory dysfunction in rats via the effect on neuroinflammation, and apoptosis, which could serve as a promising candidate for AD treatment.

## 4. Materials and Methods

### 4.1. Animals and Drug Administrations

Sprague Dawley male rats (4~5 weeks old) weighing 120~150 g were obtained from Guangdong Medical Laboratory Animal Center (Guangzhou, China) (SYXK[YUE])2018-0001) and kept in a 12-h light/dark cycle with plenty of food and water, a temperature of 22 ± 2 °C, and 40–70% relative humidity. After 2 weeks of adaptive feeding, the test animals were equally randomly divided into five groups (number = 10 rats/group). Control group: rats treated for 56 days with 0.5% (*v*/*v*) Tween 80 in 0.9% normal saline as vehicle. D-galactose group: rats treated with D-galactose (150 mg/kg/day; i.h for 56 days). Osthole-L group: rats treated with D-galactose + Osthole (10 mg/kg/day, Solvent as control, for 56 days). Osthole-H group: rats treated with D-galactose + Osthole (20 mg/kg/day, Solvent as control, for 56 days). Donepezil group: rats treated with D-galactose + Donepezil (3 mg/kg/day, Solvent as control, for 56 days). D-galactose (Aladdin Biochemical Technology, Shanghai, China) was dissolved in 0.9% saline for subcutaneous injection to induce cognitive deficits, while donepezil (Aladdin Biochemical Technology, Shanghai, China) and Osthole (purity > 98%, Tao Tao Bio, Shanghai, China) were dissolved in 0.9% saline with 0.5% (*v*/*v*) Tween 80 and administered orally for 56 days.

All animal procedures, ethics and animal welfare were approved by the Animal Ethics Committee of Guangzhou University of Chinese Medicine (No. 20210601001) and conducted in accordance with the principles and guidelines of the National Institutes of Health Guidance for the Care and Use of Laboratory Animals.

### 4.2. Behavior Analysis

#### 4.2.1. Morris Water Maze Test (MWM)

A MWM test assessed spatial learning-memory ability, including a recording system, a hidden platform (10 cm in diameter) 2 cm under the water surface, and performed in a circular pool (160 cm diameter, 60 cm height), with water temperature of 23 ± 2 °C filled to 30 cm height. Four average virtual quadrants form the pool, with the hidden platform in the heart of one. In the MWM test, rats were given adaptive training on the 1st day, followed by directed navigation on the 2nd and 6th days. During the experiment, we randomly placed each rat in the water facing the tank wall three times a day, starting from different points. An analysis of escape latency was performed based on the moment each rat found the platform from the starting point. Its escape latency would be 60 s, and it would be guided to the platform and stay there for 10 s if it could not find the platform within 60 s. The navigation in position was carried out on the 7th day of the experiment, when the rats could swim freely in 60 s without the hidden platform. For assessing spatial memory, time spent in the purpose quadrant and platform crossing times were recorded.

#### 4.2.2. Novel Object Recognition Test (NORT)

A test that measures the memory capability based on rodents’ natural ability to touch and explore new things, NORT requires a set of equipment for the experiment: including a square case (50 cm × 50 cm × 50 cm), camera equipment and VisuTrack behavior analysis software (Model: XR-VT, Shanghai Xinsoft, Shanghai, China). In the NORT test, there were two important phases: training and testing. The rats were taken to the case containing two indistinguishable objects (A and B) in opposite positions for 5 min during the training phase. Each time, the rats were placed in the same position as far as possible, with the back facing the object and at the same distance from the two objects. During testing, a fresh object with a different color and shape (object C) was substituted for one of the old objects (A). How much time the rats spent exploring the familiar and the novel object in the box within 5 min was recorded. (Putting front paws on the object, sniffing the object, licking the object, etc., are exploration objects. Posing or climbing on the object without moving cannot be considered as exploration of a new object). As a measure of objective novelty performance, the percentage of time the rat spent exploring the novel object C, out of the whole exploration time, was calculated as follows: recognition index, RI = novel object/(novel object + old object) × 100%. A 75% ethanol solution was used to clean the box and items between each test to avoid stimulating rats with odors or excrement.

### 4.3. Nissl Staining

Sodium pentobarbital was administered to four rats from each group after the behavioral experiment, and 4% paraformaldehyde was applied to perfuse brain tissues and store them for over 24 h after the perfusion. Following preservation of the brain tissues, they were immersed in 30% sucrose (4% paraformaldehyde) and dehydrated. They were then cast in embedded O.C.T., and coronal sections 20 µm thick were made by freezing microtome (LEICA CM 1950, Leica, Germany). After 10-min of Nissl staining (Beyotime Biotechnology, Shanghai, China), the sections were washed with ddH_2_O, dehydrated with gradient ethanol, made transparent with xylene and sealed with Rhamsan gum, and finally recorded with a Nikon microscope for morphology.

### 4.4. Immunofluorescence

Frozen sections were prepared in the same way as for Nissl staining. Coronal sections 20 μm thick were stained according to the steps of the Iba-1 fluorescent staining instructions. The sections were fixed with 4% paraformaldehyde for 20 min, secondarily cleaned, and sealed with 10% goat serum for 30 min. Afterward, sections were permeabilized and blocked 48 h at 4 °C with Iba-1 antibody (1:100, Abcam, Cambridge, UK). A fluorophore-conjugated secondary antibody (1:200, ZSGB-BIO, Beijing, China) was applied to the sections for 2 h, followed by Dapi (Beyotime Biotechnology, Shanghai, China) staining for an additional 10 min. ImageJ v1.8.0 software (National Institutes of Health, Bethesda, MD, USA) was used to quantify images, acquired with a fluorescence microscope Model DMi8 (Leica, Wetzlar, Germany).

### 4.5. Western Blot

Phosphatase inhibitors and protease inhibitors (Millipore, Burlington, MA, USA) were added to SDS lysis buffers to homogenize the hippocampus and cortex. With the Bradford method (Bio-Rad, Hercules, CA, USA), the protein supernatants of the lysates were obtained after centrifugation at 15,000 r and 4 °C for 15 min. Proteins were transferred to PVDF membranes (Millipore) after isolation on SDS-PAGE gels, sealed with skim milk and detected overnight with primary antibody at 4 °C. Overnight incubation occurred of endoplasmic reticulum stress related antibodies, against apoptotic antibodies and neuroinflammation antibodies: PSD95 antibody #ab12093 (abcam, Cambridge, UK); Synaptophysin antibody #ab52636 (abcam); GFAP antibody #95717 (Cell Signaling Technology(CST), Boston, MA, USA); Anti-TNF Receptor I antibody #ab19139 (abcam); Bax antibody #AF0120 (Affinity Biosciences, Cincinnati, OH, USA); Bcl-2 antibody #AF6139 (Affinity Biosciences); *β*-actin antibody # A1978 (Sigma, Ronkonkoma, NY, USA); Anti-rabbit IgG (CST). Immunoreaction of anti-mouse IgG antibody (CST) was completed, ECL luminescent solution was 1:1, and the immunoblotting was recorded by ChemiDocXRS+ (Bio-RAD, Hercules, CA, USA), a comprehensive gel imager.

### 4.6. Endophenotype Network Strategy

#### 4.6.1. Collection of Interacted Protein Targets of Osthole

The drug–target interactions (DTIs) of Osthole were first extracted from our previous integrated natural product-target database [44], which includes 38,220 high-quality experimentally validated DTIs linking 3882 natural products to 5643 human proteins. Following this, we further supplemented the targets by querying Herbal Ingredient Target Database (HIT) [45], a comprehensive database with all the herbal ingredients and target information based on literary evidence. After eliminating duplicates and non-homo sapiens targets, a total of 46 high-quality human protein targets for Osthole was received (Supplementary Material Table S1).

#### 4.6.2. Collection of Genes for Building Endophenotype Modules for AD

The endophenotype can be involved in the pathogenesis of multiple diseases at the same time, which characterizes the functional intermediate genetic characteristics of an independent biological system. Characterizing the pathogenesis of AD, building predictive models, and revealing the molecular mechanisms of AD drugs are based on constructing AD-related endophenotypic networks. In this work, we analyzed 25 sets of genes associated with several pathological AD pathological mechanisms in the Quick GO database (https://www.ebi.ac.uk/QuickGO/ (accessed on 1 March 2020)) to construct endophenotype network modules for AD.

#### 4.6.3. Construction of the Human Protein–Protein Interactome

The human protein–protein interactome served as the background against which to measure the network distance among different protein sets. Based on 15 authoritative databases, six diverse sources of protein–protein interactions (PPIs) with experimental evidence were collected: (1) protein three-dimensional (3D) interactome; (2) high-throughput Y2H binary; (3) kinase-substrate interactions; (4) signaling interactions; (5) literature protein; and (6) complexes. A total of 351,444 PPIs covering 17,706 unique proteins constitute the comprehensive high-quality human protein–protein interactome. More detailed data concerning the integration process can be found in the prior study [46].

#### 4.6.4. Network Proximity Analysis

The network proximity method was applied to analyze the relevance and the network distance between AD-related endophenotype module and Osthole’s drug targets. A gene set of endophenotype module (*A*) was performed, and a series of the drug targets (*B*), the nearest distance *d_A__B_* measured by the average shortest path length of all nodes in module *A* to module *B* in the human protein–protein interactome, defined as:

(1) $$\langle d_{AB}\rangle =\frac{1}{\left|\left|A\right|\right|+\left\|B\right\|}\left(\sum_{a\in A}{min}_{b\in B}d\left(a,b\right)+\sum_{b\in B}{min}_{a\in A}d\left(a,b\right)\right)$$

where $d\left(a,b\right)$ refers to gene a and drug target b and their shortest path length.

The significance of network distance between Osthole and AD endophenotypic modules was calculated by permutation tests with 10,000 replicates. The mean and standard deviation $\left(\sigma_{d}\right)$ of the reference distribution were used to calculate a z-score $\left(z_{d}\right)$ by normalizing the observed (non-Euclidean) distance. More information on the network proximity measure is presented in earlier studies [47,48].

### 4.7. Statistical Analysis

Statistical analysis for this study was performed by Python (V3.2). Data was analyzed using GraphPad Prism 8 (LA Jolla, San Diego, CA, USA) and presented as mean ± SEM. ImageJ was applied to calculate the strip gray value. Statistical comparisons were analyzed using one-way analysis of variance (ANOVA), followed by Bonferroni or Dunnett’s post hoc test. *p*-value < 0.05 was considered as statistically significant.

## Data Availability

Data are contained within the article and Supplementary Materials.

## Supplementary Materials

The following supporting information can be downloaded at: https://www.mdpi.com/article/10.3390/molecules29010021/s1, Table S1: Experimentally validated targets of Osthole used in this study; Table S2: Detailed information for AD-related endophenotype modules. Figure S1: Quantitative analysis of iba1 immunofluorescence.
