# Supplementary figures and images for: Network Proximity Analysis Deciphers the Pharmacological Mechanism of Osthole against D-Galactose Induced Cognitive Disorder in Rats
Source: Molecules. 2023 Dec 19;29(1):21. doi: 10.3390/molecules29010021 (PMC10779601; doi:10.3390/molecules29010021)

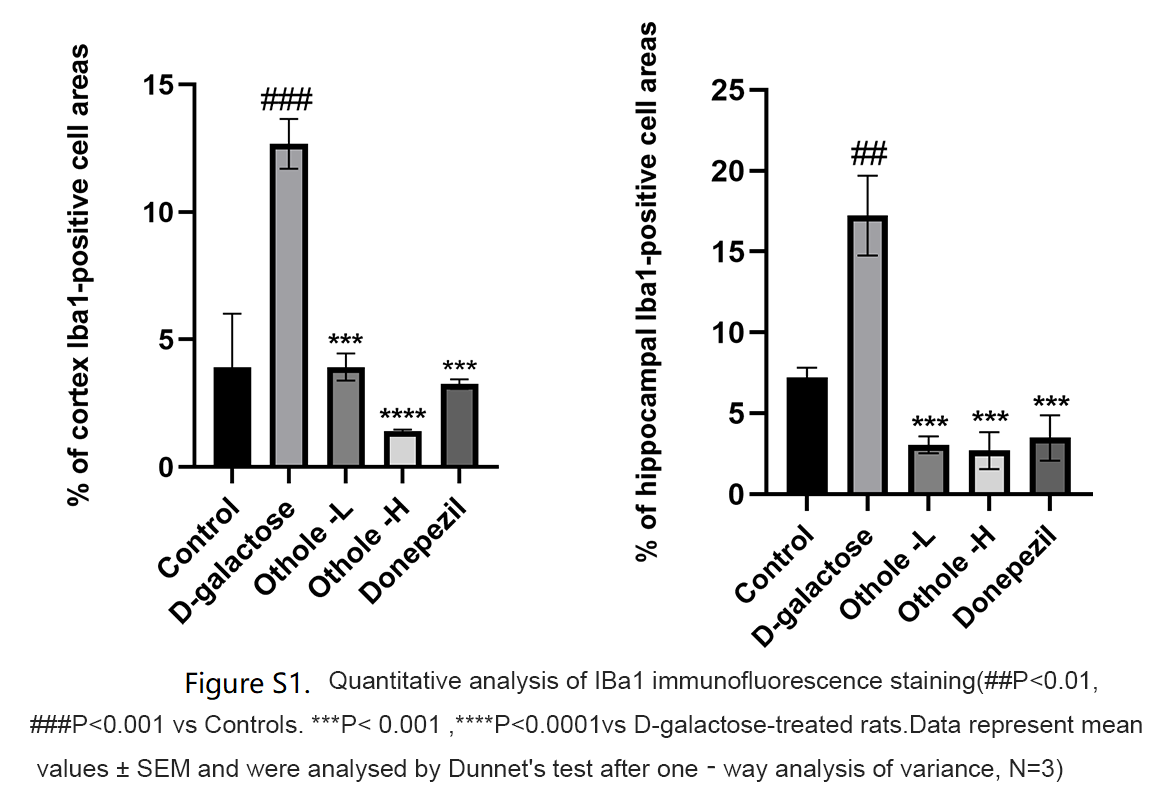

Supplement: Supplementary file 1 [file molecules-29-00021-s001.zip › Supplementary Figure S1.tif]
